# Deep Learning–Based Prediction of Refractive Error Using Photorefraction Images Captured by a Smartphone: Model Development and Validation Study

**DOI:** 10.2196/16225

**Published:** 2020-05-05

**Authors:** Jaehyeong Chun, Youngjun Kim, Kyoung Yoon Shin, Sun Hyup Han, Sei Yeul Oh, Tae-Young Chung, Kyung-Ah Park, Dong Hui Lim

**Affiliations:** 1 Department of Industrial and System Engineering Korea Advanced Institute of Science and Technology Daejeon Republic of Korea; 2 Department of Ophthalmology Samsung Medical Center Sungkyunkwan University School of Medicine Seoul Republic of Korea; 3 Department of Digital Health Samsung Advanced Institute for Health Sciences and Technology Sungkyunkwan University Seoul Republic of Korea

**Keywords:** amblyopia, cycloplegic refraction, deep learning, deep convolutional neural network, mobile phone, photorefraction, refractive error, screening

## Abstract

**Background:**

Accurately predicting refractive error in children is crucial for detecting amblyopia, which can lead to permanent visual impairment, but is potentially curable if detected early. Various tools have been adopted to more easily screen a large number of patients for amblyopia risk.

**Objective:**

For efficient screening, easy access to screening tools and an accurate prediction algorithm are the most important factors. In this study, we developed an automated deep learning–based system to predict the range of refractive error in children (mean age 4.32 years, SD 1.87 years) using 305 eccentric photorefraction images captured with a smartphone.

**Methods:**

Photorefraction images were divided into seven classes according to their spherical values as measured by cycloplegic refraction.

**Results:**

The trained deep learning model had an overall accuracy of 81.6%, with the following accuracies for each refractive error class: 80.0% for ≤−5.0 diopters (D), 77.8% for >−5.0 D and ≤−3.0 D, 82.0% for >−3.0 D and ≤−0.5 D, 83.3% for >−0.5 D and <+0.5 D, 82.8% for ≥+0.5 D and <+3.0 D, 79.3% for ≥+3.0 D and <+5.0 D, and 75.0% for ≥+5.0 D. These results indicate that our deep learning–based system performed sufficiently accurately.

**Conclusions:**

This study demonstrated the potential of precise smartphone-based prediction systems for refractive error using deep learning and further yielded a robust collection of pediatric photorefraction images.

## Introduction

Amblyopia is the most common cause of permanent visual impairment in children, and its worldwide prevalence is estimated to be approximately 1.6%-5% [1,2]. Refractive error is one of the leading causes of pediatric amblyopia [3]. Early detection of refractive error in children plays an important role in visual prognosis [4,5], and therefore, early pediatric screening is recommended by the American Academy of Pediatrics, American Academy of Pediatric Ophthalmology and Strabismus (AAPOS), and European Strabismological Association and Societies [6,7].

Cycloplegic retinoscopic refraction is the standard technique for measuring refractive error. However, this method has some limitations. It is difficult to get young children to cooperate during the procedure, and advanced clinical ophthalmologic training is required to perform the test (user dependent) [2,8].

Previously, autorefractors were developed for faster and easier refraction in children. However, autorefraction presents several difficulties, including maintaining the proper position for testing and maintaining visual fixation on the target for a sufficient duration [9,10]. Photorefraction data can confirm the presence of myopia, hyperopia, astigmatism, and anisometropia by evaluating the reflection type and the position of eccentric crescent images on the pupil after projecting a light source onto the retina [11,12]. Photorefraction is simple and fast, making it convenient for use in children with poor cooperation ability, and it is suitable for screening large populations [13,14]. Several tools have been developed to meet the growing demand to perform photorefraction in clinical settings [2,15,16]. Easy availability of these tools and accurate prediction algorithms are the most important factors for ensuring efficient screening by photorefraction. Recently, deep learning algorithms have yielded innovative results in the field of medical imaging diagnostics [17]. In particular, deep convolutional neural networks [18] have been widely applied to extract essential features directly from images without human input. In ophthalmology, deep convolutional neural networks showed remarkable performance for detecting various diseases, including diabetic retinopathy [19-21], glaucoma [22,23], and retinopathy of prematurity [24]. Deep learning can also capture biological signs that are difficult for even human experts to detect, such as retinal findings from fundus images associated with cardiovascular risk [25]. However, little research has been done on the application of deep learning to refractive error prediction among children, using photorefraction images. A previous study attempted to predict the refractive error from retinal fundus images using deep learning [26], but the application was limited because the average participant age was 55 years and a specialized device was required to obtain the fundus images.

The purpose of this study was to develop an automated deep learning–based prediction system for refractive error using eccentric photorefraction images of pediatric patients captured by a smartphone. We trained our deep convolutional neural network with photorefraction images to identify various refractive error ranges. Thereafter, we comparatively evaluated its performance on our network with conventional cycloplegic retinoscopic refraction.

## Methods

### Study Approval

This study was performed at a single center according to the tenets of the Declaration of Helsinki. The Institutional Review Board of Samsung Medical Center (Seoul, Republic of Korea) approved this study (SMC 2017-11-114).

### Participants

Patients aged 6 months to 8 years who visited the outpatient clinic for a routine ocular examination were requested to participate in this study. Written informed consent was provided by parents prior to participation. All screening tests were conducted at Samsung Medical Center between June and September 2018. The exclusion criteria were diseases that could affect light reflection, such as congenital cataracts and corneal opacity, diseases involving visual pathways or extraocular muscles, a medical history of previous ophthalmic surgery (eg, strabismus, congenital cataract, and congenital glaucoma), limited cycloplegia, and poor cooperation during study activities.

### Data Collection

A total of 305 photorefraction images (191 images from 101 girls and 114 images from 63 boys) were obtained (mean age 4.32 years, SD 1.87 years). All patients underwent a complete ophthalmologic examination, including visual acuity, motility evaluation, and anterior segment evaluation. Eccentric photorefraction images were obtained using a smartphone with a 16-megapixel camera (LGM-X800K; LG Electronics Inc, Seoul, Korea) at a 1-meter distance from the front of the patient in a dark room (<15 lux). The smartphone was placed straight forward to the face of the children without angulation. All photorefraction images were acquired in the same setting (in a dark room and before the cycloplegic procedure). The smartphone’s built-in flash, present next to the camera lens, was used as the light source for eccentric photorefraction, wherein light was refracted and reached the retinal surface and was then magnified and reflected. When optimal reflection was achieved, a characteristic crescent-shaped reflection appeared in the eye. A photograph of the crescent reflection was captured through LED control [13]. After acquisition of photorefraction images, 0.5% tropicamide and 0.5% phenylephrine (Tropherine; Hanmi Pharm, Seoul, Korea) were administered three times at 5-minute intervals. Cycloplegic retinoscopy and fundus examination to obtain spherical, cylindrical, cylindrical axis, and spherical equivalent values were performed between 30 and 60 minutes following the first instillation of cycloplegics, when the pupillary light reflex was eliminated. Both photorefraction and cycloplegic refraction were performed sequentially, and the ground truth for images acquired by photorefraction was labelled according to the values of cycloplegic refraction. Consequently, the result of cycloplegic refraction was provided as the ground truth for machine learning of photorefration images.

The acquired eccentric photorefraction images were divided into the following seven classes according to the spherical values measured by cycloplegic refraction: ≤−5.0 diopter (D), >−5.0 D and ≤−3.0 D, >−3.0 D and ≤−0.5 D, >−0.5 D and <+0.5 D, ≥+0.5 D and <+3.0 D, ≥+3.0 D and <+5.0 D, and ≥+5.0 D. The cutoff values of the seven classes for refractive errors were determined clinically. Among myopic refraction (minus values), −5.0 D, −3.0 D, and −0.5 D were considered as thresholds of high, moderate, and mild myopia, respectively. In other words, refractive errors ≤−5.0 D indicated high myopia, refractive errors >−5.0 D and ≤−3.0 D indicated moderate myopia, and refractive errors >−3.0 D and ≤−0.5 D indicated mild myopia. Similarly, +0.5 D, +3.0 D, and +5.0 D were thresholds of mild, moderate, and high hyperopia, respectively, among plus values.

### Image Data Preparation for Training, Validation, and Testing

Photorefraction images were processed for training our deep convolutional neural network. Initially, the images were cropped to capture the pupil. The images were resized to 224×224 pixels, and the pixel values were scaled from 0 to 1. To overcome an overfitting issue caused by an insufficiently sized training dataset, data augmentation was performed by altering brightness, saturation, hue, and contrast; adding Gaussian noise; and blurring images using Gaussian kernels. Thereafter, the image pixel values were normalized by subtracting the mean and dividing by the SD to ensure that each image had a similar data distribution and would converge faster during the training procedure.

For training, validation, and testing, we used the five-fold cross-validation approach to build a reliable deep learning model with a limited dataset. Initially, all the data were subdivided into five equal-sized folds with the same proportion of different classes in each fold. Four of the five folds were for training and validation (3.5 folds for training and 0.5 folds for validation), and one fold was for testing. After five repetitions of this process, we were able to evaluate the performance of the entire dataset because the test folds were independent of each other, and we confirmed the stability of our model for the entire dataset using the confusion matrix.

### Deep Convolutional Neural Network and Training

**Figure 1:**
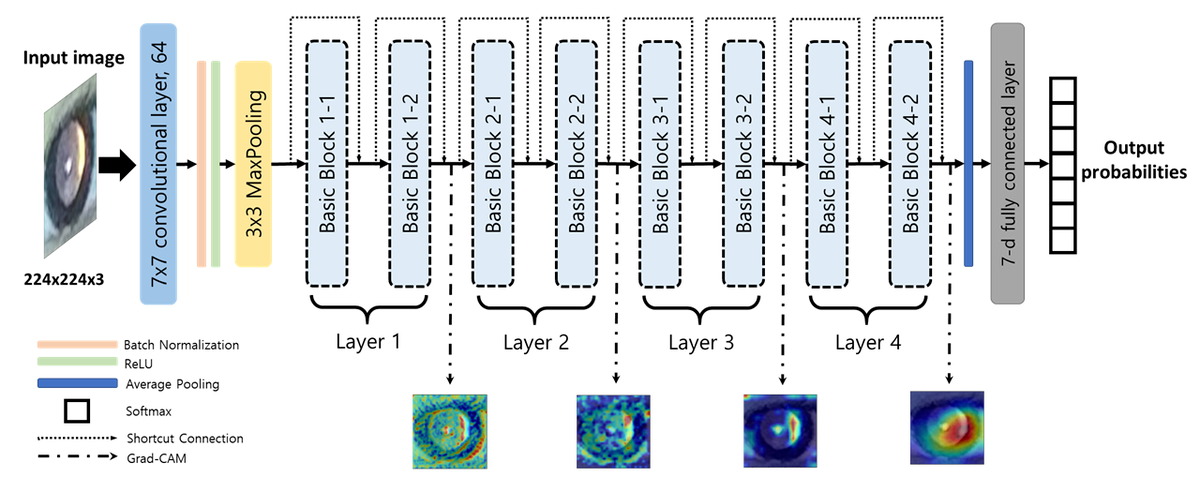
Overview of the proposed deep convolutional neural network architecture. The photorefraction image inputs pass through 17 convolutional layers and one fully connected layer, and the outputs of the network assign the probabilities for each refractive error class given the image. We also generate the localization map highlighting the important regions from the final convolutional feature maps of the layer i (i=1, 2, 3, or 4).

**Figure 2:**
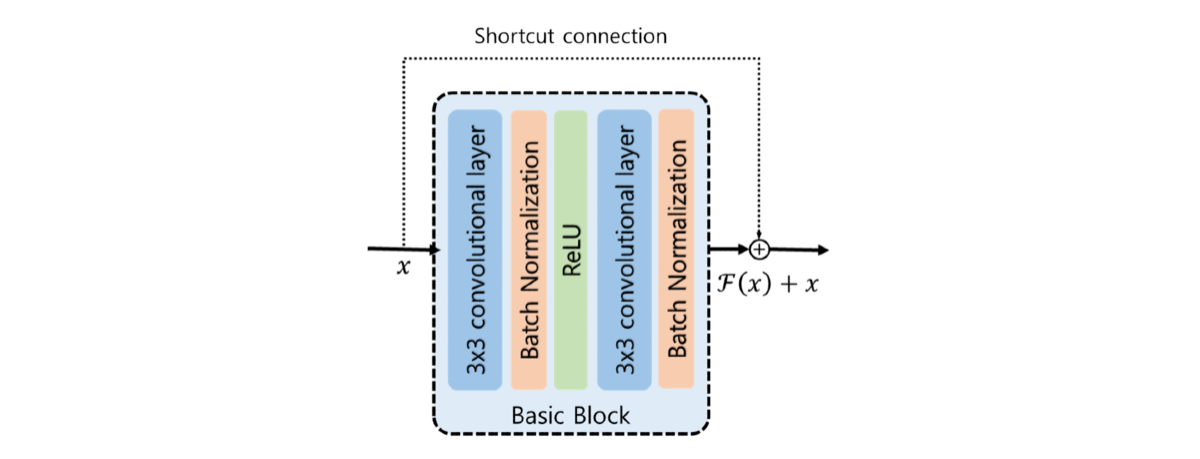
Structure of the basic block and the shortcut connection. The basic block consists of two 3×3 convolutional layers, two Batch Normalization layers, and a Rectified Linear Unit (ReLU) activation function. The shortcut connection adds the input vector of the basic block to the output of the basic block.

We used a deep convolutional neural network to classify photorefraction images into the most probable class of refractive error. Among the various types of convolutional neural networks, we developed Residual Network (ResNet-18) [27] to avoid problems that occur when deep neural network depth increases, such as vanishing or exploding gradients and accuracy degradation. Residual Network addresses these issues using identity mapping with shortcut connections. The shortcut connections allow networks to skip over layers and also enable speed training. Figure 1 illustrates the overall structure of the deep learning approach we propose in this work. The basic block consists of two 3×3 convolutional layers, and the shortcut connection enables the network to learn identity mapping (Figure 2).

Because we did not have a sufficiently large training dataset, we performed transfer learning to capture low-level features, such as edge and color, without wasting image data [28]. Accordingly, pretrained parameters of Residual Network on the ImageNet [29] datasets were reused as starting points for our model. The pretrained Residual Network was available on Pytorch [30]. We then replaced the last fully connected layer to output seven predicted probabilities for each refractive error class (≤−5.0 D, >−5.0 D and ≤−3.0 D, >−3.0 D and ≤−0.5 D, >−0.5 D and <+0.5 D, ≥+0.5 D and <+3.0 D, ≥+3.0 D and <+5.0 D, and ≥+5.0 D). During the training process, the first layer was frozen, and the learning rates for the subsequent layers were increased from 1e-10 to 1e-5 to finetune our network for preventing an overfitting issue. Furthermore, we designed the loss function as a weighted sum of cross-entropy by class, wherein the weight for each class was the reciprocal of the proportion of that class’s images in the training dataset. This technique was useful to achieve balanced accuracy for all classes, despite having an imbalanced training dataset. For convergence of network training, the learning rate was decayed by a factor of 0.95 every 10 epoch, and we trained the parameters of networks using stochastic gradient descent [31] with 0.9 momentum. We set the maximum training epoch as 500 and the minibatch size of training images as 16. All codes were implemented using Pytorch 1.2.0 [30]. Details of the network structure are shown in Table 1.

**Table 1:** Configuration of the deep convolutional network.

Layer type, feature map			Filters	Kernel	Stride	Padding		Learning rate
**Input**								
	224×224×3		—^a^	—	—	—		0.0 (freeze)
**Convolutional**								
	112×112×64		64	7×7×3	2	3		0.0 (freeze)
**Batch normalization**								
	112×112×64		—	—	—	—		0.0 (freeze)
**Max pooling**								
	56×56×64		1	3×3	2	1		0.0 (freeze)
**Layer 1**								
	**Basic block 1-1**							
		56×56×64	64	3×3×64	1	1		0.0 (freeze)
		56×56×64	64	3×3×64	1	1		0.0 (freeze)
	**Basic block 1-2**							
		56×56×64	64	3×3×64	1	1		0.0 (freeze)
		56×56×64	64	3×3×64	1	1		0.0 (freeze)
**Layer 2**								
	**Basic block 2-1**							
		28×28×128	128	3×3×64	2	1		1e-10
		28×28×128	128	3×3×128	1	1		1e-10
		28×28×128	128	1×1×64	2	0		1e-10
	**Basic block 2-2**							
		28×28×128	128	3×3×128	1	1		1e-10
		28×28×128	128	3×3×128	1	1		1e-10
**Layer 3**								
	**Basic block 3-1**							
		14×14×256	256	3×3×128	2	1		1e-8
		14×14×256	256	3×3×256	1	1		1e-8
		14×14×256	256	1×1×128	2	0		1e-8
	**Basic block 3-2**							
		14×14×256	256	3×3×256	1	1		1e-8
		14×14×256	256	3×3×256	1	1		1e-8
**Layer 4**								
	**Basic block 4-1**							
		7×7×512	512	3×3×256	2	1		1e-6
		7×7×512	512	3×3×512	1	1		1e-6
		7×7×512	512	1×1×64	2	0		1e-6
	**Basic block 4-2**							
		7×7×512	512	3×3×512	1	1		1e-6
		7×7×512	512	3×3×512	1	1		1e-6
**Average pooling**								
	1×1×512		1	7×7	7	0		—
**Fully connected layer**								
	1×7		—	—	—	—		1e-5
**Softmax**								
	1×7		—	—	—	—		—

^a^Not applicable.

## Results

### Image Dataset Demographics

A total of 305 photorefraction images from 191 girls and 114 boys were acquired. The mean age was 4.32 years (SD 1.87 years), and the median age was 4 years (range 0-8 years). The mean spherical equivalent was 0.13 D (SD 2.27 D; range −5.50 to 6.75 D), and the mean astigmatism was −1.50 D (SD 1.38 D; range −6.50 to 0 D), according to cycloplegic refraction.

**Figure 3:**
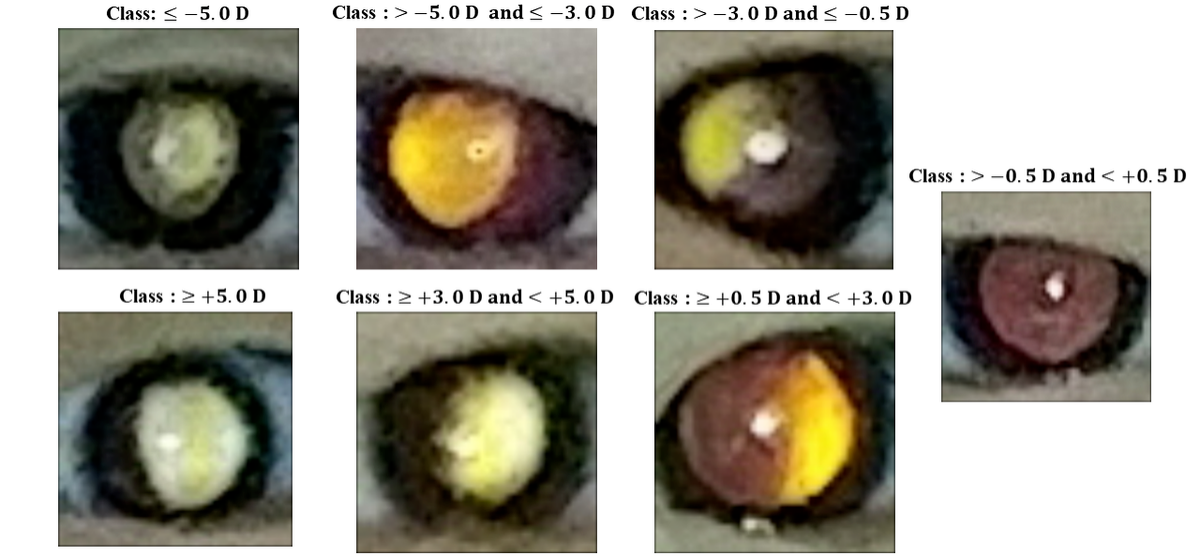
Examples of photorefraction images from the seven different refractor error classes. A bright crescent appears in the pupillary reflex, and its size and shape indicate the diopter (D) value.

According to cycloplegic refraction results, 25 photorefraction images had a refractive error ≤−5.0 D, 18 had an error >−5.0 D and ≤−3.0 D, 50 had an error >−3.0 D and ≤−0.5 D, 84 had an error >−0.5 D and <+0.5 D, 87 had an error ≥+0.5 D and <+3.0 D, 29 had an error ≥+3.0 D and <+5.0 D, and 12 had an error ≥+5.0 D. Table 2 summarizes patient demographics in detail, and examples of photorefraction images according to the refractive error class are shown in Figure 3.

**Table 2:** Dataset participant demographics.

Characteristic		Value
Total images, n		305
**Refractive error, n**		
	≤−5.0 D^a^	25
	>−5.0 D and ≤−3.0 D	18
	>−3.0 D and ≤−0.5 D	50
	>−0.5 D and <+0.5 D	84
	≥+0.5 D and <+3.0 D	87
	≥+3.0 D and <+5.0 D	29
	≥+5.0 D	12
Girls, n (%)		191 (62.6)
Age, mean (SD)		4.32 (1.87)

^a^D: diopters.

### Performance of the Proposed Deep Convolutional Neural Network

We used five-fold cross-validation to evaluate our network’s performance. Training, validation, and testing were independently iterated five times. In each iteration, there were 213 training images, 31 validation images, and 61 testing images. We chose the network with the highest validation accuracy when loss of training was saturated. Thereafter, we measured the classification accuracy of the network in the test fold. All five networks, which were established in the training phase, had an accuracy of more than 80% for each validation set. Similarly, the performances of the five testing folds were 83.6%, 80.3%, 82.0%, 78.7%, and 83.6% (Table 3).

**Table 3:** Results for five-fold cross-validation.

Iteration^a^	Validation accuracy (%) (N=31)	Test accuracy (%) (N=61)
First iteration	87.1	83.6
Second iteration	80.6	80.3
Third iteration	80.6	82.0
Fourth iteration	83.9	78.7
Fifth iteration	83.9	83.6
Average	83.2	81.6

^a^In each iteration, our network was trained using the rest of the validation and test dataset (213 training images).

In the five-fold test, our network had the following accuracies: 80.0% for class ≤−5.0 D, 77.8% for class >−5.0 D and ≤−3.0 D, 82.0% for class >−3.0 D and ≤−0.5 D, 83.3% for class >−0.5 D and <+0.5 D, 82.8% for class ≥+0.5 D and <+3.0 D, 79.3% for class ≥+3.0 D and <+5.0 D, and 75% for class ≥+5.0 D (Table 4). Despite the imbalanced dataset, our model achieved consistent performance for all classes.

In addition, our network maintained the stability of prediction for refractive error, as shown in the confusion matrix (Table 5). Overall, 85.7% (48/56) of total misclassifications were within one class difference and 98.2% (55/56) of total misclassifications were within two class differences.

**Table 4:** Performance of our deep convolutional neural network with the overall test dataset.

Class	Number	Accuracy (%)
≤−5.0 D^a^	25	80.0
>−5.0 D and ≤−3.0 D	18	77.8
>−3.0 D and ≤−0.5 D	50	82.0
>−0.5 D and <+0.5 D	84	83.3
≥+0.5 D and <+3.0 D	87	82.8
≥+3.0 D and <+5.0 D	29	79.3
≥+5.0 D	12	75.0
Total	305	81.6

^a^D: diopter.

For performance comparison, we developed the following five baseline models and calculated the performances: (1) pretrained VGG-11 [32]; (2) pretrained squeezeNet [33]; (3) Support Vector Machine (SVM) [34]; (4) Random Forest [35]; and (5) simple convolutional neural network. VGG-11 and squeezeNet were pretrained on the ImageNet [29] datasets, and their parameters were frozen, except the last four convolutional layers during training. Moreover, we designed the following two traditional machine learning approaches: SVM and Random Forest. SVM has a radial basis function kernel, 1.0 regularization parameter, and three degrees of the kernel function. Random Forests has 500 trees, the Gini index criterion, and two samples required to split an internal node. Lastly, the simple convolutional neural network has three convolutional layers with six kernels (8×8size, two strides), 16 kernels (5×5size, two strides), and 24 kernels (3×3 size, one stride), respectively; a max-pooling layer (2×2 size and two strides) after each convolutional layer; and three fully connected layers with 120, 84, and 7 hidden units, respectively, in a row at the end of the network. We evaluated the performances of the five baseline models using five-fold cross-validation, and the results of performance comparison are shown in Table 6. We confirmed that the proposed deep convolutional neural network outperformed all baseline models.

**Table 5:** Confusion matrix for refractive error classification of our deep convolutional neural network.

Ground truth	Predictive value							Accuracy (%)	
	≤−5.0 D^a^	>−5.0 D and ≤−3.0 D	>−3.0 D and ≤−0.5 D	>−0.5 D and <+0.5 D	≥+0.5 D and <+3.0 D	≥+3.0 D and <+5.0 D	≥+5.0 D		
≤−5.0 D	20^b^	3	2	0	0	0	0		80.0
>−5.0 D and ≤−3.0 D	1	14^b^	2	0	1	0	0		77.8
>−3.0 D and ≤−0.5 D	1	4	41^b^	4	0	0	0		82.0
>−0.5 D and <+0.5 D	0	0	5	70^b^	8	1	0		83.3
≥+0.5 D and <+3.0 D	0	0	1	10	72^b^	4	0		82.8
≥+3.0 D and <+5.0 D	0	0	0	1	4	23^b^	1		79.3
≥+5.0 D	0	0	0	0	1	2	9^b^		75.0
Overall accuracy (%)	—^c^	—	—	—	—	—	—		81.6

^a^D: diopter.

^b^Number of correct predictions of our deep convolutional neural network.

^c^Not applicable.

**Table 6:** Performance comparison of the proposed model and baseline models.

Model	Accuracy (%)
The proposed deep convolutional neural network	81.6
Pretrained VGG-11	70.8
Pretrained SqueezeNet	77.4
Support Vector Machine	65.2
Random Forest	62.9
Simple convolutional neural network	70.8

**Figure 4:**
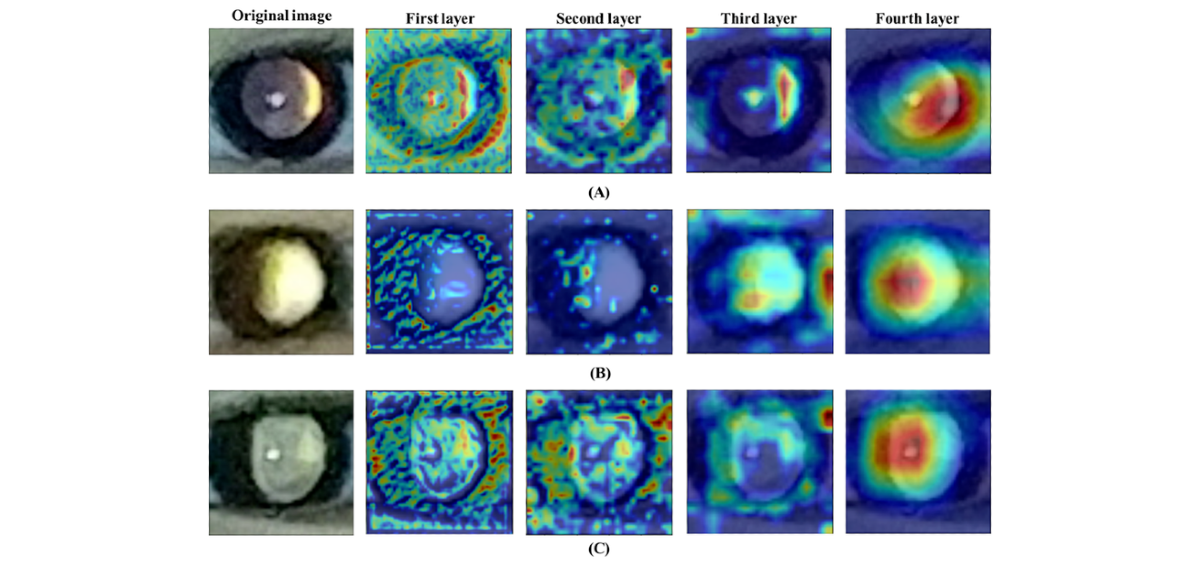
Examples of photorefraction images correctly classified by deep neural networks. (A), (B), (C) were identified as ≥+0.5 D and <+3.0 D, ≥+3.0 D and <+5.0 D, and ≥+5.0 D, respectively. The first layers captured low-level features, such as edge and color. With deeper layers, the network focused on high-level features that were regarded as important aspects for classification.

Additionally, we produced heatmaps using gradient-weighted class activation mapping (Grad-CAM) [36] to provide visual explanations for each screening decision. This technique is crucial for interpreting network output and validating whether the network learned meaningful features. The activation map visualizes where the network considered the critical locations to be within photorefraction images for detecting refractive error. Figure 4 shows the activated regions from four layers in the photorefraction images. Notably, we observed the heatmap from the fourth layer, which captured important features for classifying refractive error, particularly the region of the crescent in the pupil.

## Discussion

The primary purpose of refractive error screening is the early detection of a refractive error to allow interventions that can reduce the risk of amblyopia. Early detection and treatment of refractive error can lead to better visual outcomes and reduce the prevalence and severity of amblyopia in children [4,37]. The cycloplegic refraction test has been an essential tool to accurately measure refractive error, because pediatric patients are more accommodating than adults [38]. However, young children tend not to cooperate well during the refraction test, and the test requires a skilled ophthalmic practitioner [2,8]. Additionally, the eye drops used during cycloplegia can cause side effects, such as flushing, fever, drowsiness, and red eye [39]. For these reasons, cycloplegic refraction is not suitable for large screening of refractive error and amblyopia [12]. Currently, smartphones are ubiquitous devices that allow physicians and other related medical professionals to overcome common diagnostic barriers in many clinical settings [40]. A photorefraction screening test using a smartphone is an easy and effective way to screen most young children. The photorefractive method is simple and takes no longer than a second to test both eyes simultaneously. The test requires minimal space (just a meter of distance between the subject and the testing device) and removes the need for cycloplegia, thereby greatly reducing side effects and testing time. Moreover, it does not require expert knowledge or experience to perform [6]. These advantages make the photorefractive method ideal for measuring refractive error, especially for poorly cooperative young children.

Several studies have compared the accuracy of photoscreeners for detecting various amblyopia risk factors [40-42]. One study evaluated a smartphone photoscreening application (GoCheckKids) and reported 76% sensitivity and 67.2% specificity [15] for detecting amblyopia risk factors using the 2013 AAPOS guidelines. Because we evaluated the accuracy of predicting refractive errors and not amblyopia risk factors, we were limited in our ability to directly compare the performance of our method against that of GoCheckKids. Instead, our deep convolutional neural network achieved satisfactory accuracy for predicting categories of refractive error using only a small image dataset. The results showed the potential for developing precise smartphone-based prediction systems for refractive error using deep learning. With further collection of pediatric photorefraction image data, more precise prediction of refractive error and effective detection of amblyopia would be possible.

This study compared refractive error estimation with precycloplegic photorefraction images and cycloplegic refraction. The results showed consistent measurements between the two methods. Dubious results regarding estimation of refractive error using photorefractors have been uncovered by previous studies [12,14,42]. Erdurmus et al reported that noncycloplegic photorefraction (Plusoptix CR03; PlusoptiX GMBH, Nurnberg, Germany) tended to overestimate negative refraction in children, resulting in overdiagnosis of myopia (−0.70 D) [12]. Lim et al reported similar results and showed that refractive error measured by a photorefractor without cycloplegia (PlusoptiX S09; PlusoptiX GmbH) tended to be more myopic compared with cycloplegic refractive error [42]. On the other hand, Schimizek et al claimed that noncycloplegic refraction using a photorefractometer (Power Refractor; PlusoptiX GmbH) resulted in underestimation of spherical equivalents owing to uncontrolled accommodation [14]. Another study showed that cycloplegic refraction results and photorefractor Plusoptix S08 (Plusoptix GmbH, Nurnberg, Germany) results were similar [2]. In this study, photorefraction results without cycloplegia showed reasonable agreement with cycloplegic refraction, suggesting that our deep learning–based system achieved considerably accurate performance under noncycloplegic conditions.

This study has several limitations. First, manifest refraction was not performed in all subjects. Since photorefractive refraction tests were performed without the use of a cycloplegic agent, useful information might have been obtained if the number of manifest refraction results without cycloplegia were enough to compare with photorefraction data in the same patient. Second, the number of photorefraction images was relatively small and the model could only predict a range of refractive errors (not a specific value). Third, all children involved in the study were Korean. Thus, a trained model using the eyes of Korean children may not be applicable to the eyes of pediatric patients having different ethnicities [43,44]. Future studies with more patients of multiple ethnicities and a greater range of refractive errors would be beneficial for providing a more precise clinical perspective.

In conclusion, this study showed that our deep learning–based system successfully yielded accurate and precise refractive measurements. This further demonstrates the potential for developing simplified smartphone-based prediction systems for refractive error using deep learning with large-scale collection of pediatric photorefraction images from patients with various ages and refractive errors.

## Abbreviations

AAPOS: American Academy of Pediatric Ophthalmology and Strabismus
SVM: Support Vector Machine
